# Helicobacter pylori infection and osteoporosis in elderly patients

**Published:** 2015

**Authors:** Behzad Heidari

**Affiliations:** 1 Mobility impairment research center, Babol University of Medical Sciences, Babol, Iran; 2 Department of Internal Medicine, Ayatollah Rouhani Hospital, Babol University of Medical Sciences, Babol, Iran.

Osteoporosis is one major cause of bone fracture and subsequent morbidity and mortality in the elderly population (1). Several clinical and demographic parameters including aging, menopause, parity, inflammatory disease, hormonal, gastrointestinal, renal and metabolic disorders like vitamin D deficiency, obesity, weight loss may affect bone mass and result in bone mineral density changes (BMD) and osteoporosis (2-6). 

Helicobacter pylori (H.pylori) which is a known cause of gastritis and peptic ulcer disease is associated with systemic inflammation. In addition, H.pylori infection has been linked to many other disorders like autoimmune thyroid diseases, diabetes mellitus, dyslipidemia, obesity, osteoporosis and primary hyperparathyroidism (7).

In this issue of the journal, Fotouk Kiai et al. have investigated the relationship between H.pylori infection and osteoporosis in the elderly population of Amirkola Health Ageing Project (AHAP) and the results indicated no significant differences in BMD between subjects with and without H.pylori infection (8). This context was investigated in several previously published studies with conflicting results (9-11). Lin et al. in a study of Taiwanese female patients with upper gastrointestinal diseases found an association between H. pylori infection and osteoporosis (1). Kim et al. demonstrated that the presence of atrophic gastritis was associated with increased osteoporosis by odds ratio of 1.89 (11). In contrast, in a study by Kakehasi et al. in patients with gastritis, the risk of osteoporosis in postmenopausal women did not increase due to atrophic gastritis or H.pylori infection (10). Similarly, in an earlier study by us, H.pylori infection was not associated with osteoporosis (12). Figura et al. in one study demonstrated that in male patients with osteoporosis H.pylori CagA-positive infection was prevalent and the level of estrogen was lower and bone turnover was greater (13). Explanation of the results regarding H.pylori infection and osteoporosis is associated with several limitations. 

1-H. pylori infection in older patients is prevalent and a positive serum antibody against H. pylori cannot confirm an active systemic inflammation. In addition, seropositivity alone in elderly subjects without any dyspeptic syndrome cannot be considered as an active systemic inflammation.

2- Several factors may also affect bone mass and make it impossible to determine the independent association between H. pylori infection and osteoporosis.

3-In patients with H.pylori infection many associated factors including dyspepsia, administration of proton pump inhibitors, changes in diet, and disorder in calcium absorption (14) may affect bone mass and confound the results

4-Patients’ older age, menopause, previous pregnancies and lack of physical activity due to knee osteoarthritis (3, 15-17) are the most common causes of BMD changes. Distribution of these factors may be different across the study groups and so affect the results.

5- Although the patients with inflammatory disorders were excluded from the study, but the presence of a number of undiagnosed patients at earlier stage of inflammatory arthritis like rheumatoid arthritis should not be ignored. These patients are at greater risk of bone loss (5, 6, 18, 19). Many patients like chronic obstructive pulmonary disease and chronic renal disease are associated with inflammation (20-22) and so are at greater risk of bone loss.

6-Diagnosis of osteoporosis based on the BMD T-score in older patients may underestimate the real prevalence of osteoporosis. Since osteoarthritis is prevalent in older subjects and the presence of osteophytes results in falsely elevation of BMD and underestimation of osteoporosis particularly at the spine (11).

7- Distribution and the severity of osteoarthritis may differ between the comparison groups and so the patients with and without H.pylori infection may be affected differently thus, the results are expected to be confounded.

8- This study was a cross-sectional and case-control in which the results do not indicate causality. 

9-High level of serum antibody indicates the past history of H.pylori infection. So, the duration of active infection and its contribution in the development of osteoporosis is difficult to be determined

These observations indicate that the relationship between H.Pylori infection and BMD changes requires a longitudinal study of patients with active systemic inflammation which has been confirmed by rapid urease test or by gastric biopsy. Nonetheless, treatment of patients with dyspepsia and active inflammation can affect the impact of H.pylori on BMD changes and confound the results.
